# An agent-based model to investigate microbial initiation of Alzheimer’s via the olfactory system

**DOI:** 10.1186/s12976-020-00123-w

**Published:** 2020-04-15

**Authors:** Shalini Sundar, Carly Battistoni, Ryan McNulty, Fernando Morales, Jonathan Gorky, Henry Foley, Prasad Dhurjati

**Affiliations:** 1 Department of Chemical and Biomolecular Engineering, University of Delaware, Newark, Delaware USA; 2 Department of Pathology, Anatomy, and Cell Biology, Thomas Jefferson University, Philadelphia, PA USA; 3 New York Institute of Technology, New York, NY USA

**Keywords:** Agent-based model, Biological modelling framework, Alzheimer’s disease, Olfactory system, NetLogo3-D, Microbiome

## Abstract

**Background:**

Alzheimer’s disease (AD) is a degenerative brain disease. A novel agent-based modelling framework was developed in NetLogo 3D to provide fundamental insights into the potential mechanisms by which a microbe (eg. *Chlamydia pneumoniae*) may play a role in late-onset AD. The objective of our initial model is to simulate one possible spatial and temporal pathway of bacterial propagation via the olfactory system, which may then lead to AD symptoms. The model maps the bacteria infecting cells from the nasal cavity and the olfactory epithelium, through the olfactory bulb and into the olfactory cortex and hippocampus regions of the brain.

**Results:**

Based on the set of biological rules, simulated randomized infection by the microbe led to the formation of beta-amyloid (Aβ) plaque and neurofibrillary (NF) tangles as well as caused immune responses. Our initial simulations demonstrated that breathing in *C. pneumoniae* can result in infection propagation and significant buildup of Aβ plaque and NF tangles in the olfactory cortex and hippocampus. Our model also indicated how mucosal and neural immunity can play a significant role in the pathway considered. Lower immunities, correlated with elderly individuals, had quicker and more Aβ plaque and NF tangle formation counts. In contrast, higher immunities, correlated with younger individuals, demonstrated little to no such formation.

**Conclusion:**

The modelling framework provides an organized visual representation of how AD progression may occur via the olfactory system to better understand disease pathogenesis. The model confirms current conclusions in available research but can be easily adjusted to match future evidence and be used by researchers for their own individual purposes. The goal of our initial model is to ultimately guide further hypothesis refinement and experimental testing to better understand the dynamic system interactions present in the etiology and pathogenesis of AD.

## Background

Alzheimer’s Disease (AD) is a progressive, degenerative disease of the brain associated with dementia, mood instability and cognitive impairment. Currently, approximately 5.8 million Americans have been diagnosed with AD: 5.6 million individuals of age 65 and older and 200,000 individuals younger than age 65 [1]. More specifically, AD affects 3% of the population between 65 and 75 years of age and 47% of the population over 85 years [2]. The U. S National Institute of Health (NIH) attributes a cost of over $100 billion per year to AD and estimates that, with the current state of population dynamics, Alzheimer’s prevalence may triple within the next 30 years due the aging baby boom generation [3]. In consequence, there have been significant research efforts to diagnosis, treat and possibly prevent the disease progression. As of 2019, the U. S Food and Drug Administration (FDA) has approved six drugs to alleviate symptoms of AD [1]. In 2012, The National Alzheimer’s Plan was released to outline an approach for more effective prevention methods by 2025 [4]. However, despite such efforts, AD is not completely understood, and treatment modalities have yet to yield remission or halt progression. This has, in turn, motivated comprehensive investigations into new avenues of research such as the use of personalized biomarker tests [1].

One supported conceptualization on what may trigger AD proposes that fibrous protein aggregates, beta-amyloids (Aβ), initiate disease pathogenesis [5]. Aβ peptides consist of 40–42 amino acids and result from mis-cleavage of the amyloid precursor protein (APP). Normal APP cleavage by α-secretase and γ-secretase results in smaller peptide units that can be digested properly by the body [6]. However, in an AD patient, APP is cleaved by β-secretase and γ-secretase, causing Aβ aggregates to form [6]. Extracellular Aβ aggregates can cause mechanical disruption of signaling, activate microglial immune responses, increase the risk of hemorrhagic stroke and initiate intracellular hyperphosphorylation of the tau protein [1]. The tau protein plays a fundamental role in the structural support of microtubules and hyperphosphorylation of the tau protein separates it from the microtubule, leading to neuronal death. These separated tau protein molecules aggregate to then form what are known as neurofibrillary (NF) tangles [6]. Aβ plaque and NF tangles have been associated with cognitive impairment and the pathology of AD [7, 8]. It has also been found that the earliest concentrations of these molecules and earliest brain damage occur in the entorhinal cortex and the hippocampus regions of the brain, leading to AD’s hallmark symptom: short-term memory loss [2].

Recent studies have demonstrated that bacterial or viral infections may possibly initiate the generation and deposition of Aβ plaque and NF tangles in the brain. These infections have been shown to lead to AD related symptoms. For example, research found that Herpes Simplex Type 1 virus (HSV1), *Chlamydia pneumoniae* and Spirochetes, directly caused Aβ plaque to form in mice and cell culture samples [9]. Specifically, for *C. pneumoniae*, the bacteria may bypass the blood brain barrier by travelling through the olfactory system: from the nasal cavity into the olfactory system and spread through regions of the brain including the entorhinal cortex and hippocampus [10–12]. In fact, bacterial initiation may be linked to the abnormal gene expression seen in AD. One study found that *C. pneumoniae* possibly causes the dysfunctional AD calcium-related gene expression: human neuronal cells injected with the microbes exhibited similar gene expression as brain cells from AD patients [13]. However, it is important to note that AD may not have a single cause but is rather a result of multiple factors [1].

We have developed an agent-based computational framework in NetLogo 3D to model the possible precursor steps wherein *C. pneumoniae* may lead to late-onset AD development via the olfactory system [14]. Although there are existing AD models (such as agent-based, network and mathematical), our work may be the first to model AD pathogenesis from a microbial perspective [15–17]. As described in this paper, the model demonstrates how a direct bacterial infection and propagation may occur and how immunity can play a significant role in that older patients demonstrate quicker Aβ plaque and NF tangle production. Its ability to describe the environment in a flexible, visual and simplified manner can make it immensely helpful in understanding more about AD progression. Ultimately, our novel model provides the foundation for evaluating one possible pathway of what we refer to as the *C. pneumoniae*-Olfactory mechanism and provides guidance for future hypothesis formation/refinement and experimental testing.

### The *C. pneumoniae-*olfactory mechanism

*C. pneumoniae* is a pathogenic, obligate intracellular bacteria that has distinct, parasitic effects throughout the human body [12]. It exhibits a biphasic life cycle and has as an incubation period on the order of weeks making it difficult to detect early and treat [18]. During one phase of its life cycle, *C. pneumoniae* takes the form of an elementary body (EB), which is responsible for infection of host cells. This form is metabolically inactive and attaches to and enters the host, generally through phagocytosis. The second form of *C. pneumoniae* is known as a reticulate body (RB), which is the non-infectious form of the bacteria. An RB is metabolically active and replicates through binary fission once inside of an endosome within the host cell. Upon replicating, RBs reorganize back into EBs, to be released back into the system environment via cell lysis, extrusion or exocytosis [12]. There have been two suggested lengths for this life cycle. The first life cycle takes 72 h from EB infection, EB to RB transformation, RB replication, RB to EB reconversion, and excretion of EB. The second suggests that the bacteria can remain in an RB state for longer periods of time. It was found, however, that the shorter life cycle was exhibited by infected microglia and epithelial cells [10]. This shorter life cycle was used in the development of our model. Furthermore, EBs cannot survive outside a host cell environment for long periods of time as they rely on the enzymatic machinery of the host to satisfy many metabolic requirements [19]. However, persistence may allow a small fraction of the microbial population to survive for long periods of time, potentially causing recurring infection and can be a potential untested confounder for the model as highlighted in the discussion of this paper [20].

**Fig. 1:**
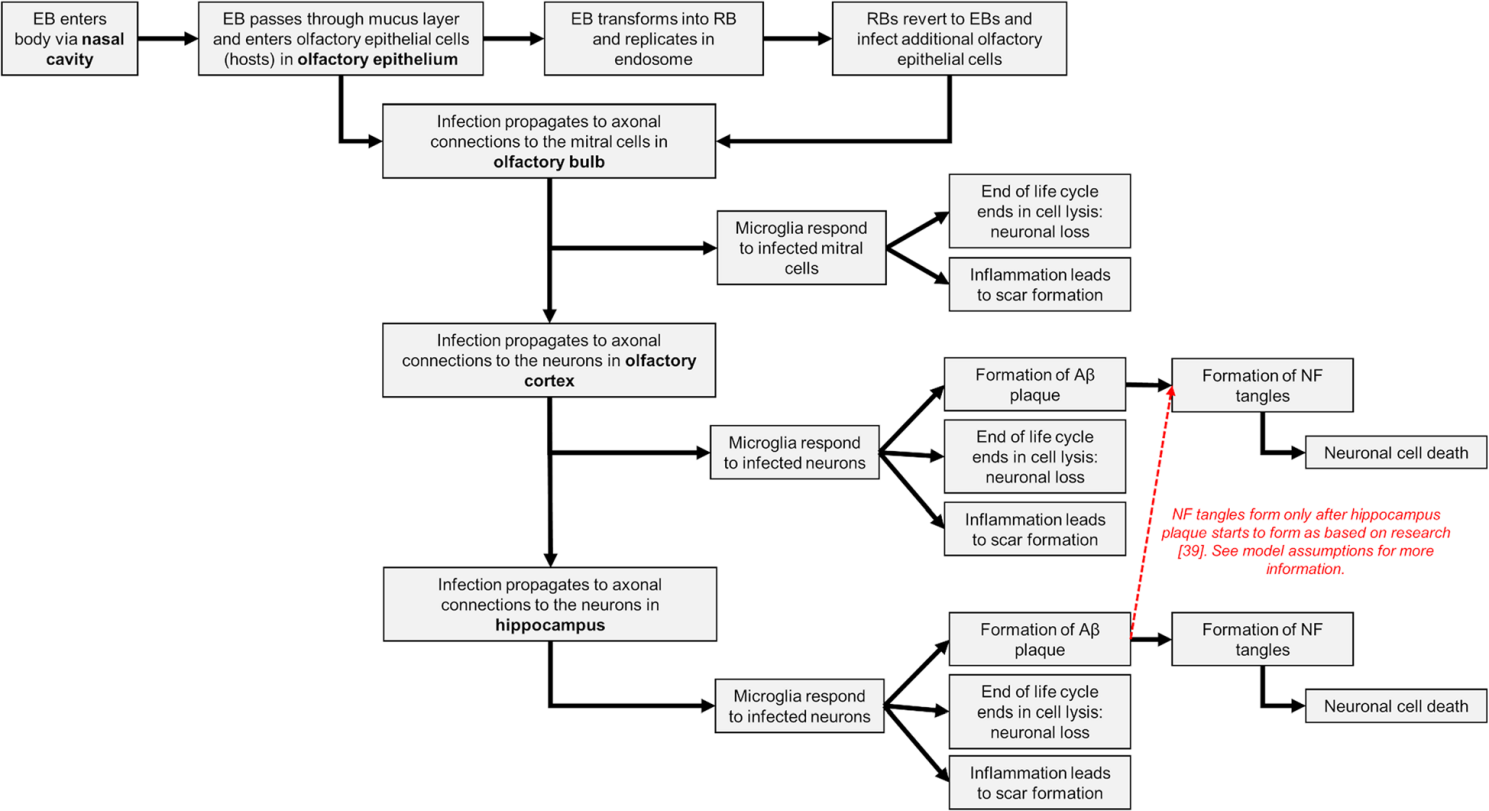
Connectivity model of *C. pneumoniae*-Olfactory mechanism used in the NetLogo model*.* Proposed outline of *C. pneumoniae* infection propagation and effects as adapted from mouse study described in Little et al. [11]

Studies have demonstrated that there may be a link between *C. pneumoniae* and Aβ plaque deposition in mice as well as humans [10–12] [21–23]. Available work suggests that *C. pneumoniae* travels via the olfactory system: from the nasal cavity, through the olfactory bulb and into parts of the brain which receive smell information such as the entorhinal cortex and hippocampus [10–12, 23]. As the microbes pass through these regions, they may initiate Aβ plaque production and evoke immune responses [10]. Based on this hypothesis and the associated studies, one possible pathway for the *C. pneumoniae*-Olfactory mechanism was mapped in our NetLogo 3D model (Fig. 1).

Initial evidence suggests that the link between *C. pneumoniae* and AD Aβ plaque formation may be causative. It has been argued that the pathogenic nature of the microbe might elicit immune responses, triggering AD pathogenesis [9]. For example, once the infectious agent reaches the central nervous system, it may lie dormant until reactivated by a waning immune system resulting from aging [9]. The formation of Aβ plaque is just one of the defense mechanisms against microbial reactivation [24]. Additionally, biopsies of AD patients have revealed microbial concentrations in the entorhinal cortex and hippocampus [11, 21, 22]. These regions of the brain exhibited both *C. pneumoniae* immunoreactivity as well as Aβ plaque and NF tangle formation [23, 25]. Current understanding of AD suggest that the entorhinal cortex and hippocampus are among earliest regions to be damaged in late-onset AD [26, 27]. Taken together, these observations suggest a relationship between AD and *C. pneumoniae* and may implicate *C. pneumoniae* as a factor in the initiation of AD as opposed to an incidental occurrence in AD tissue samples.

## Method

### Model overview

Our model was developed in the NetLogo 3D (v6.0.1), an agent-based modeling was developed at Northwestern University’s Center for Connected Learning and Computer-Based Modelling (https://ccl.northwestern.edu/netlogo). The approach taken in our model development mirrors the “top-down” approach often used in systems modelling. In this approach, a model is developed by starting from the larger system level and adding detail as needed to account for system complexities [28].

**Fig. 2:**
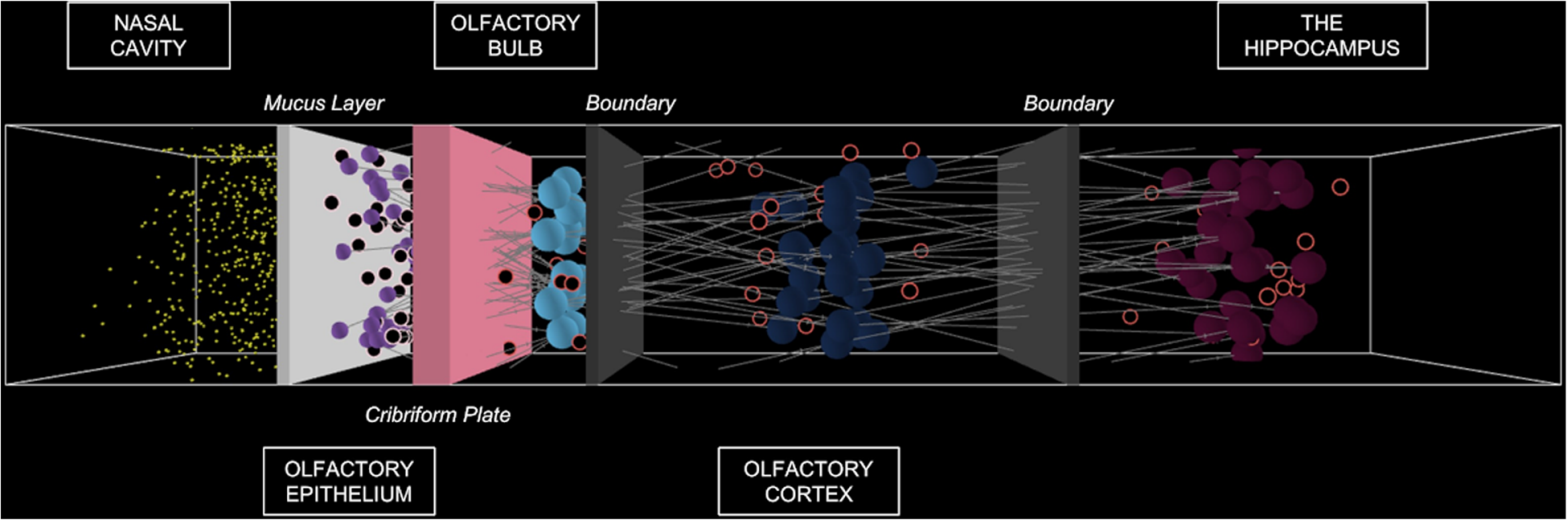
3D spatial view of NetLogo model simulation. Regions of interest have been labelled. From left to right: the nasal cavity, the olfactory epithelium, the olfactory bulb, the olfactory cortex and the hippocampus

In the direct infection pathway considered in the model, the microbe travels from an outside environment through the nasal cavity and olfactory bulb and into the brain regions that receive smell information. A simulation view upon startup of the program model is depicted in Fig. 2. The spatial arrangement of the studied system is separated into five regions of interest: the nasal cavity, the olfactory epithelium, the olfactory bulb, the olfactory cortex and the hippocampus.

The results of simulations are presented in a variety of real time plots. For example, one plot displays the number of infected sustentacular cells, olfactory epithelium cells, mitral cells, olfactory cortex neurons and the hippocampus neurons. Other plots display real time counts of total Aβ plaque patches and NF tangle x’s separately formed in the olfactory cortex and hippocampus. The relevant variables, cell concentrations and rates of cellular processes, used in the NetLogo model are listed in Table 1. All the variables are adjustable via sliders on the user interface and recommended values are provided in the user interface. Adjustable variable settings also allow the user to test multiple combinations of the initially set parameters to see the impact of these variables under different scenarios. However, recommended variable values, reflecting those found in prior art literature and our initial model testing, have been provided to guide the user.

**Table 1:** Key variables used to adjust the system environment of the model

**Concentrations**	
EBs of *C. pneumoniae* bacteria in outside environment	
Olfactory cells in olfactory epithelium	
Sustentacular cells in olfactory epithelium	
Mitral cells in olfactory bulb	
Microglia in olfactory bulb	
Neurons in olfactory cortex	
Microglia in olfactory cortex	
Neurons in hippocampus	
Microglia in hippocampus	
**Rates**	
Olfactory cell recovery rate	
*C. pneumoniae* chance of infection of olfactory cells	
Microglia repair distance	
Olfactory cell division	
Replication of reticulate bodies in endosome of hosts	
Infected cell lysis	

### Model legend

The cells present in each model region are listed in Table 2. Additionally, colored barriers in the NetLogo simulation distinguish the different compartments of the model. The white barrier represents the mucus layer between the nasal cavity and olfactory epithelium. The light pink barrier represents the cribriform plate between the olfactory epithelium and olfactory bulb. The gray barriers distinguish between the remaining regions of interest, namely the olfactory cortex and hippocampus.

**Table 2:** NetLogo Model Agent Legend

Model Agent	Intended Designation
Small yellow spheres	EBs of C. Pneumoniae
Tan/pink edged circles	Sustentacular cells in the olfactory epithelium
Filled purple spheres	Olfactory epithelium cells in the olfactory epithelium
Filled light blue spheres	Mitral cells in the olfactory bulb
Red edged cells	Microglia in the olfactory bulb, olfactory cortex and hippocampus
Filled dark blue cells	Neurons in the olfactory cortex
Filled magenta cells	Neurons in the hippocampus
White patches	Aβ plaque
Orange X’s	NF tangles

Additionally, the following parameters were used to appropriately scale the model. Through relative ratios of the thicknesses of the olfactory epithelium, cribriform plate and olfactory bulb, the model better represents a virtual human patient.

- Incubation period of C. pneumoniae: 1–3 weeks [14]
- Thickness of olfactory epithelium: ~ 150 μm [29]
- Thickness of olfactory bulb: ~ 0.55 cm [30]
- Thickness of cribriform plate: ~ 0.55 cm

The different cells sizes in the model have been tabulated in Table 3. Again, ratios of cell sizes were used to appropriately scale the simulation to realistic dimensions. Neurons in the olfactory cortex and hippocampus were assumed to be the same size as mitral cells since there is limited available knowledge as to what exact cell type in these regions are affected. More information about this specific simplification is included in the list of model rules.

**Table 3:** Cell sizes used to accurately scale NetLogo model [11, 29, 31, 32]

Cell Type	Size (μm)
*C. pneumoniae* (EB)	0.2–0.4 [11]
Olfactory Epithelial	3–5 [29]
Sustentacular	3–5 [29]
Mitral	10.1 [31]
Olfactory Cortex Neurons	Assumed to be same as Mitral
Hippocampus Neurons	Assumed to be same as Mitral
Microglia	4.6 [32]

### Model assumptions

As a starting point, it was necessary to simplify the pathway through which *C. pneumoniae* has been hypothesized to enter the body and travel into the brain [11, 33]. The simplified pathway was a direct infection mechanism through which *C. pneumoniae* enters the nasal cavity of the host from the outside environment and travels through the olfactory epithelium, as based on the findings of Little et al. [11]. It is important to note that the considered pathway may allow the obligate intracellular bacteria to enter the brain without passing through normal defenses such as the blood brain barrier, which typically resists microbial invasion. In one study, mice were inoculated intranasally with *C. pneumoniae (*just as a patient in our model breathes in the microbe) and observed over the course of 3 months [11]. Through light and electron microscopy, there was evidence of infection presence in the olfactory epithelium and olfactory bulb. After a month into the study, Aβ fibers were found in the olfactory bulbs of the mice [11].

Based on this work and the outline described in Fig. 1, it was assumed that the virtual patient is exposed to *C. pneumoniae*, via their nasal cavity, which begins to make its way through the mucus barrier and into the olfactory epithelium. In the olfactory epithelium, if an EB microbe is within infecting distance of a cell, it will infect the cell, represented by changing the cell color to green. Upon infection of the olfactory epithelium cells, it was assumed that the bacteria would reproduce and spread to other cells through axonal connections. As a result, the infection can spread through the links between connecting cells through the olfactory bulb, olfactory cortex and hippocampus. As a side note, research has demonstrated *C. pneumoniae* disseminates rapidly through a silent infection strategy in the lungs, taking advantage of neutrophil apoptosis and the clearance mechanism of phagocytes to move between cells [21]. As in reality, multiple olfactory epithelium cells are connected to a single mitral cell and it was assumed that the infection of each connecting olfactory cell and its subsequent nerve fiber was needed to effectively infect a mitral cell with *C. pneumoniae*. Mitral cells are then connected to neuronal cells in the olfactory cortex which are subsequently connected to neuronal cells in the hippocampus. The infection travels to different cells and regions as soon as all the in-links of a cell are infected and turned green. In the olfactory epithelium, olfactory cortex and hippocampus, microglia activate an immune response to heal infected cells. However, in the olfactory cortex and hippocampus only, healing of a neuron causes a white Aβ plaque patch to form. Only after Aβ plaque patches form in the hippocampus, orange NF tangle x’s begin to form in either the olfactory cortex or the hippocampus based on the mechanistic behavior. Aβ plaque patches form near infected cells and NF tangles form randomly in the specific region. Additionally, only one Aβ plaque patch forms per healing of an infected cell and a small number of NF tangles form per healing of the infected cell. These amounts of production were chosen arbitrarily due to limited available knowledge but can be changed easily with future research.

Additionally, entities with complexities not substantially relevant for our model were lumped together [28]. For example, the olfactory system exhibits widespread connectivity [34]. To reduce this complexity, physical representations of the olfactory cilia, glomeruli, olfactory tract and olfactory (bowman’s) glands were excluded from our model. However, some of the main biological roles of these components were incorporated into the depiction and behavior of included species by lumped analysis. The role of the olfactory cilia, which are the first to communicate/interact with outside agents (such as *C. pneumoniae* EBs) was placed within the olfactory cells themselves. This behavior is demonstrated whenever the olfactory cells, which move randomly throughout the epithelial layer, move near the mucus layer wall and are now susceptible to becoming infected. The function of basal cells, or stem cells with the capability to create both olfactory and sustentacular cells, was incorporated into the code through the “reproduce” function which introduces new olfactory cells into the olfactory epithelium at a set frequency. Lastly, the function of the glomeruli, which connect olfactory cell axons to the dendrites of mitral cells was lumped into each “link” present in the system, providing a pathway for infection to travel into the olfactory bulb of the brain. This concept is a critical aspect of the *C. pneumoniae*-Olfactory mechanism as it rationalizes the possible presence of the bacteria in regions of the brain that may be directly associated with the olfactory tract such as the entorhinal cortex and the hippocampus [21, 22].

### Model rules

In our model, the following set of specific rules were assigned to individual cells and are continuously looped as the simulation runs.

- **There is random movement of cells within each region representing interactions between cells.** While a realistic illustration of cell motion within the body would be quite restrained due to the packed nature of bone, tissue, etc., the model aimed to represent the widespread interactions between cells, achieved mainly through cell signaling (e.g. autocrine, paracrine, and juxtacrine). This rule encouraged the spread of bacteria upon infection, not only in terms of olfactory cells but also along their axonal “links”. Ultimately, this leads to distributed Aβ plaque and NF tangle formation within the brain upon long-term bacterial exposure and infection propagation.
- **There is directional flow of*C. pneumoniae*through nasal cavity.** The EBs of *C. pneumoniae* that were modeled to pass through the nasal cavity (left most region of environment) were assigned to move primarily in the positive to negative y-direction. This simulated the expected flow of the bacteria through the system upon breathing in. In addition, the cells could vary slightly in the x and z directions to induce a more random walk which allows some of the bacteria to encounter the mucus layer of the nasal cavity. When in contact with this layer, the agents were designed to move more slowly in all directions, simulating the viscoelastic nature of this region.
- **EBs of*C. pneumoniae*bacteria have short lifespans outside the host cell.** The bacterial lifespan can be adjusted from the model interface, however EBs of *C. pneumoniae* do not persist very long when outside of a host cell [21].
- **RBs of*C. pneumoniae*bacteria replicate into x number of EBs.** This number x can be adjusted on the interface of the model to match findings from literature. The preset value was chosen to be 20, based on the outcomes of multiple preliminary simulations with varied initial conditions.
- **There is total olfactory cell linkage to mitral cells.** Axon formation greatly influences the survival rate of olfactory cells within the olfactory epithelium [35]. Based on this and for increased model simplicity, all olfactory cells were created with immediate “links” to mitral cells. The sustentacular cells do not contain any axon links. It is important to note that links displayed in the model often appear to jump around the screen. This is due to the implicit behavior of links in NetLogo which constantly try to minimize the link length between agents. Ultimately, this behavior does not affect the model as the links remain assigned to their original cells, providing a virtual connection for infection spread.
- **Infection of olfactory epithelium cells occurs when in very close proximity to EBs.** The infecting distance can be adjusted directly from the interface of the model and represents the EBs of *C. pneumoniae* coming into “contact” with the olfactory epithelial cells which are used as hosts. This assumption was made due to the limited knowledge of cell to cell transfer of obligate intracellular bacteria [21]. Sustentacular cells, which do not connect to mitral cells, were exempt from infection to simplify the code and reduce the ease in which bacteria could propagate (early simulations found rapid infection rates).
- **Infection of an olfactory epithelium cell causes immediate infection of its axon.** This was one of the broader assumptions made in the modeling framework that can be improved as more literature is available. Mainly, it simplifies the pathway and infection mechanism which connects olfactory cells to mitral cells. Essentially, this rule assumes that the rate of bacterial infection of olfactory cells is the limiting step in the infection mechanism. Once an olfactory cell becomes infected, it was assumed that infection of the connected nerve cells (which make up the axon which travels through the cribriform plate to intertwine with the dendrites of a mitral cell in the glomeruli) is inevitable and rapid. In the model, this rapid spread of infection from the olfactory epithelium to the olfactory bulb was represented through the links, which turn green as soon as their connected olfactory cells would turn green. As stated in the previous rule, the mechanism of infection for obligate intracellular bacteria from an adjacent cell is unknown [21].
- **Olfactory cells without “links” die.** As found in literature, olfactory cells without axons have a much lower survivability rate [35]. For this reason, the code eliminates any olfactory cells without links. Since the only agent this rule affects are infected olfactory cells whose connecting mitral cells were just renewed, this rule essentially states that all infected olfactory cells connecting to a repaired mitral cell die.
- **Cells become fully infected once all connected input cells are infected.** It was decided that cells would only become infected upon infection of all their connecting “in-links” – representing the infection of all the axons which interact directly with the dendrites of the connecting cells. This rule reduces the rapid rate of cell infection that was initially occurring in preliminary simulation testing. As a result, this rule assumes that cells will have the maximum potential for infection once all their connected cells, from which they receive sensory input, are infected.
- **Repaired cells lose original “links” to connecting input cells.** To simplify the system further, it was assumed that repair of mitral cells in the olfactory bulb, neuronal cells in the olfactory cortex and neuronal cells in the hippocampus leads to the degradation of all infected axons which are linked to a specific cell. This rule was made to circumvent the lack of detail regarding axon/dendrite mechanism while avoiding the oversimplification that cell repair leads to total pathway repair.
- **Each mitral cell is linked to neurons in a lumped olfactory cortex.** Since the olfactory system has various pathways for transfer of smell information to the hippocampus, a lumped olfactory cortex was used as the next region after the olfactory bulb. This accounts for the entorhinal cortex, where *C. pneumoniae* has been found in AD brain samples, as well as other parts of the olfactory system where the infection could possibly travel before reaching the hippocampus [21, 22]. Connections from the olfactory cortex project from mitral cells [36]. For further simplification, the cells in this region are not specified and referred to as just neuronal cells since there is limited knowledge as to what exact regions and cells of the olfactory cortex are affected in the bacterial infection.
- **Each olfactory cortex neuronal cell is linked to neuronal cells in the hippocampus.** The olfactory cortex project directly to the neuronal cells in the hippocampus region of the model. The olfactory cortex sends direct input to the hippocampus from the entorhinal cortex to account for smell information in centers of the brain for learning and behavior [37]. Again, since there is limited knowledge as to what exact parts and cells of the hippocampus are affected in the bacterial infection, these cells are referred to as neuronal cells as a simplification. However, inclusion of the hippocampus in the model is important as it accounts for the microbial concentrations found in this region of the brain in AD brain samples [21, 22].
- **Microglia swarm all infected cells in the olfactory bulb, olfactory cortex and hippocampus.** While the microglia are modeled to move randomly about these regions when the system is in normal state, the infection of a cell “activates” these defense cells causing them to flock towards all sources of infection with the simulation. This rule demonstrates the effect of cell signaling in biological systems as the glial cells change their behavior based on information passed out by other cells in the environment. Their motility is reliant on filamentous actin to propel the microglia to the source of the anomaly [32]. An interesting consequence of enacting this rule in the code was that, upon the simultaneous infection of multiple cells, the microglia were forced to split their time between infection sites – thus decreasing the chance of cell repair.
- **Microglia renew infected cells in close proximity for the prolonged duration of the simulation.** This replaces the infected cell with healthy cell (represented by coloring the cell back to its original color in the simulation), once the microglia fell within a certain specified distance of the infected cell. This distance can be adjusted from the interface of the simulation and represents effective “contact” between a microglial cell and an infected cell.
- **Fully infected neuronal cells in the olfactory cortex and neuronal cells in the hippocampus cause systematic amyloid plaque and random neurofibrillary tangle formation, progressively damaging the brain.** Following the main tenet of the *C. pneumoniae* hypothesis, it was modeled that infected cells within these regions would cause Aβ plaque and NF tangle formation to form in the specified region. Since it has been found that immune response cells aggregate around areas where amyloid plaque formation by several studies [25, 38], areas where microglia swarm an infected cell produces a single patch of plaque in the model. Since there is limited knowledge as to how tangle formation is initiated in a similar way, tangles are formed randomly in the model within the specified region. Additionally, however, further research may be needed to specify more accurate numbers of formation, but the model can be easily modified to fit the data.
- **Amyloid plaque formation occurs first and then tangle formation occurs.** In mice, it has been found that Aβ plaque form before NF tangles do in Alzheimer’s [39]. This was accounted for the model by forcing NF tangles to form only after Aβ plaque begins to deposit in the hippocampus. Since there is limited knowledge on the time between the Aβ plaque production and NF tangle production, for the purposes of our model, NF tangles begin to form immediately after Aβ plaque begin to form in the hippocampus.

## Results

The “Go” button should be selected to run the simulation. EBs of *C. pneumoniae* can be introduced into the system by flipping the C-Pneumoniae-Exposure switch to “on”. The bacteria in the outside environment passes from the nasal cavity, through the mucus barrier and into the olfactory epithelium. The EBs travel mainly in the y-direction with slight variation in x and z-directions. The bacterial flux in the nasal cavity can be varied as the simulation continues to run to model various bacterial exposure types. Infection then propagates to the olfactory bulb, olfactory cortex and hippocampus regions. Additionally, NetLogo does not run in normal time but through “ticks”, so that each tick represents a single iteration or loop through the code. For the purposes of our model, a proportion of 100 ticks to 1 day was predetermined to facilitate the application/relation of model output to behavior observed in real systems in real time. This proportion was used in all simulation runs.

### Response to constant microbial flux

**Fig. 3:**
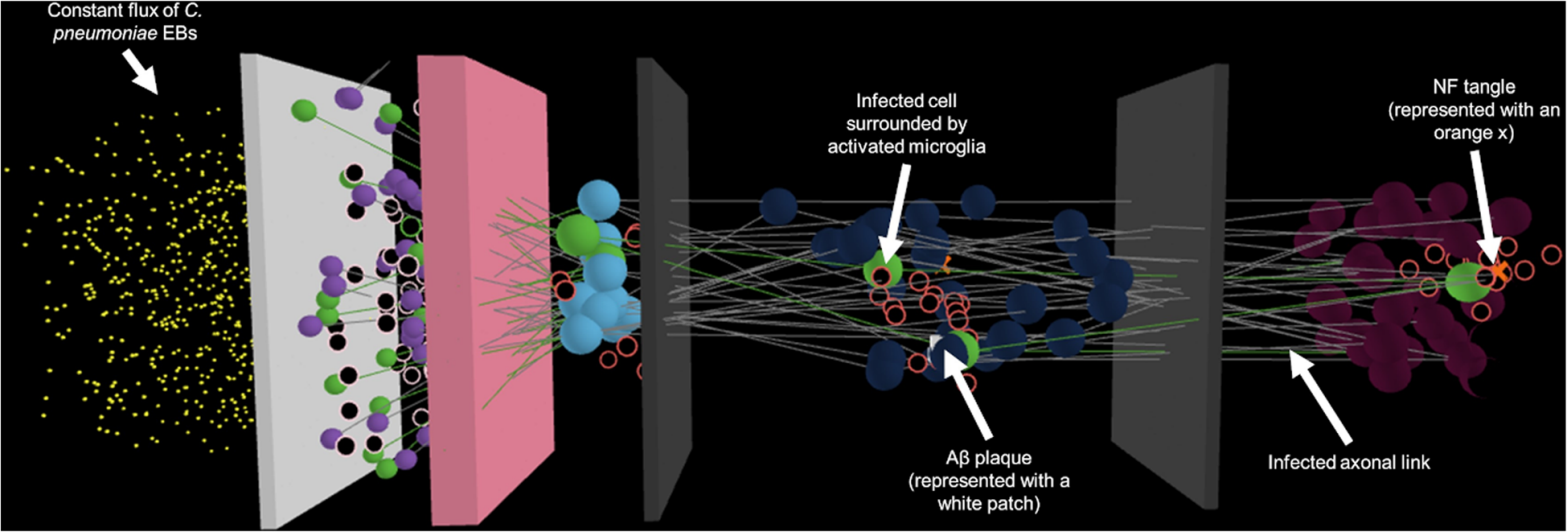
NetLogo 3D simulation view after exposure to a constant flux of *C. pneumoniae* (time equivalent ~ 24 days). Plaque begins to form around 23/24 days (olfactory cortex/hippocampus) and this simulation was used as a baseline for time scales and concentrations in subsequent simulations

One observation from running multiple simulations is that, even with a constant flux of *C. pneumoniae* bacteria through the nasal cavity, there is a delay for when the infection propagates enough to cause Aβ plaque formation. This is demonstrated in Fig. 3, which shows that for constant flux of a large microbial concentration, it still takes roughly a month for any Aβ plaque to form. In the simulation run in Fig. 3, Aβ plaque (white patches) start forming after approximately 23 days in the olfactory cortex and after approximately 24 days in the hippocampus. This simulation was run as a baseline for determining the timeframe and initial microbial concentration for subsequent simulation runs.

### Response to altered immunity

**Fig. 4:**
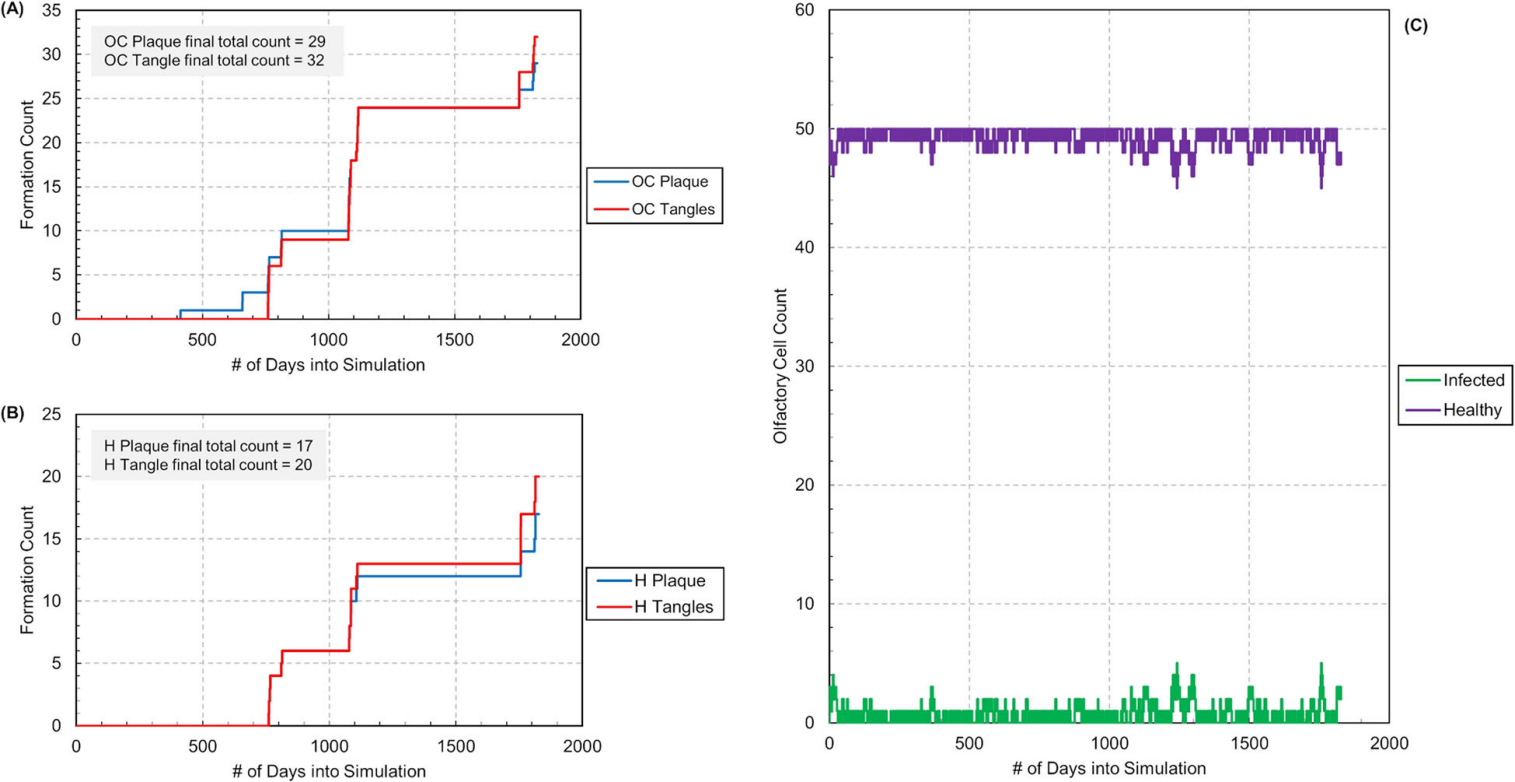
Simulation results for low olfactory recovery ability of 0.3 (time equivalent ~ 5 years). **a** Plot of Aβ plaque and NF tangle counts in the olfactory cortex (OC) versus simulation run time in days. **b** Plot of Aβ plaque and NF tangle counts in the hippocampus (H) versus simulation run time in days. **c** Plot of infected and healthy olfactory cell counts in the olfactory epithelium versus simulation run time in days

**Fig. 5:**
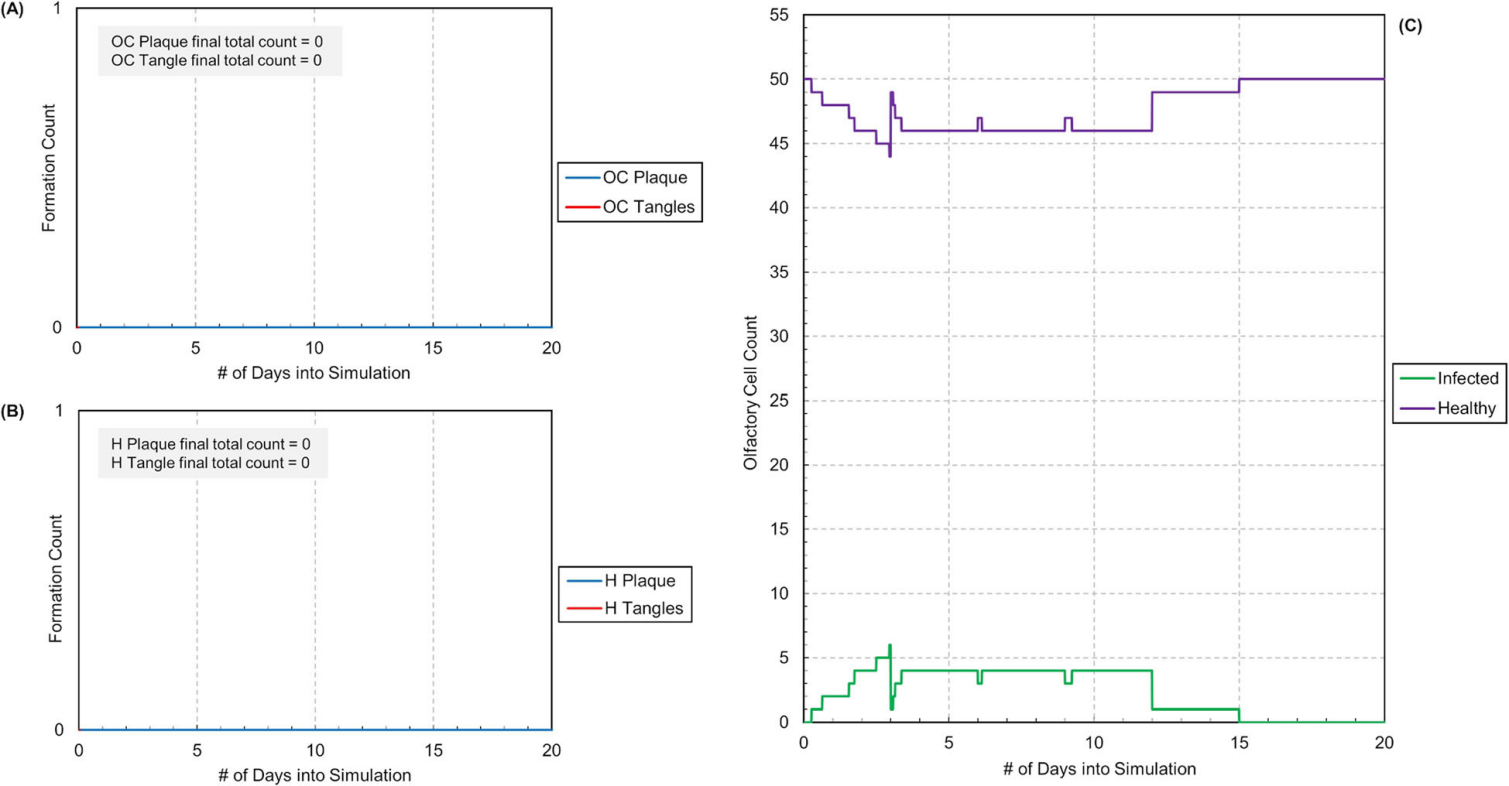
Simulation results for high olfactory recovery ability of 0.7 (time equivalent ~ 5 years). Infection ends after approximately 15 days. **a** Plot of Aβ plaque and NF tangle counts in the olfactory cortex (OC) versus simulation run time in days. **b** Plot of Aβ plaque and NF tangle counts in the hippocampus (H) versus simulation run time in days. **c** Plot of infected and healthy olfactory cell counts in the olfactory epithelium versus simulation run time in days

To test more realistic situations, the *C. pneumoniae* exposure was then limited to approximately 100 to 300 ticks or roughly 1 to 3 days to represent more realistic exposure time frames. We decided to test the effects of varied immunities, using the olfactory recovery ability variable in the simulation. Low and high olfactory recovery abilities were chosen to test and compare simulation results (see Figs. 4 and 5). The lower olfactory recovery ability was used to represent the lower immunity an older individual might have. The higher olfactory recovery ability was used to represent the more robust immunity a younger individual might have. Both simulations were run for 185,000 ticks, or just over 5 years, to test the model over a long-time frame. Additionally, a large number for the initial EB concentration was used to reduce simulation time for Aβ plaque buildup, however adjusting the EB microbial concentration may be needed in future model testing/refinement.

The simulation results over time show that enough exposure to *C. pneumoniae* in the nasal cavity as well as increased ability for infection propagation can lead to substantial buildup of Aβ plaque and NF tangles in the olfactory cortex and hippocampus regions of the brain. We see that with the lower recovery ability, the first plaque started forming in the olfactory cortex around 40,000 ticks or roughly 1 year into the simulation. The first plaque started forming in the hippocampus around 80,000 ticks or roughly 2 years into the simulation. While this might seem very early, it is important to note that individuals only start exhibiting AD symptoms after approximately 20 years of significant Aβ plaque buildup [1]. There is a delay after initial Aβ plaque deposition wherein the brain will initially atone for any damage until it cannot do so anymore due to significant damage [1]. Additionally, research suggests the AD may occur in three stages: a pre-clinical stage where patients exhibit AD biomarkers but not its symptoms, a mild cognitive impairment stage where patients exhibit symptoms that do not affect their day to day activities and finally, a dementia stage [1]. As a result, while the simulations demonstrate very early Aβ plaque and NF tangle formation, these results seem to be in accordance with typical AD progression rates. In contrast, for the higher recovery ability, no Aβ plaque and NF tangle form in the olfactory cortex or hippocampus within the 5 years. After the initial dip in healthy olfactory cells, the simulations demonstrate that the body can fight off infection and maintain an absolute steady state, which occurs around 15 days. This seems consistent with the fact that AD does not usually develop at younger ages (perhaps due to a more robust immune systems).

Furthermore, in the lower recovery ability, it was seen that infection seemed to follow in spurts, probably since infection propagates through axonal connections in our model. Once a mitral cell was infected, the infection spread to both the olfactory cortex and hippocampus causing the Aβ plaque and NF tangle counts to shoot up rapidly. It was also interesting to see delays between these infection spurts. These delays probably exist as links “die” after cells are healed by microglia. As a result, it is difficult for the infection to propagate very fast through the connections between remaining cells. We also found that our simulations mirrored experimental results from literature in that Aβ plaque production almost always formed in the olfactory cortex before the hippocampus as the simulation ran or as the virtual patient became “older” [39]. Typically, NF tangle formation began in the hippocampus and then progressed to the olfactory cortex as suggested by experimental data, however this was not always the case [39].

In the lower recovery simulation, the model converges on a steady state between the number of infected and healthy olfactory cells despite Aβ plaque and NF tangle formation. Within the model, this may be due to a balance between the healthy olfactory cell reproduction rate and infected cell lyse rate. This may be attributed to an equilibration between the rates at play - olfactory production, olfactory repair and cell lysis.

Table 4 lists the initial conditions under which these simulations were run. All other settings not listed in this table (e.g. olfactory cell tails, bacteria tails, *C. pneumoniae* exposure) were held constant between each trial.

**Table 4:** Settings of user selected parameters for altered immunity simulations (Figs. 4 and 5)

Attribute	Fig 4 Values	Fig 5 Values
EB Concentration	100	100
Initial Number of Olfactory Cells	50	50
Initial Number of Sustentacular Cells	50	50
Initial Number of Mitral Cells	15	15
Initial Number of Microglia (Olfactory Bulb)	15	15
Initial Number of Olfactory Cortex Neurons	30	30
Initial Number of Microglia (Olfactory Cortex)	15	15
Initial Number of Hippocampus Neurons	30	30
Initial Number of Microglia (Hippocampus)	15	15
Chance of Infection	1.0	1.0
Microglia Chance of Healing	0.2	0.2
**Olfactory Recovery Ability**	**0.3**	**0.7**

### Response to altered chance of infection

**Fig. 6:**
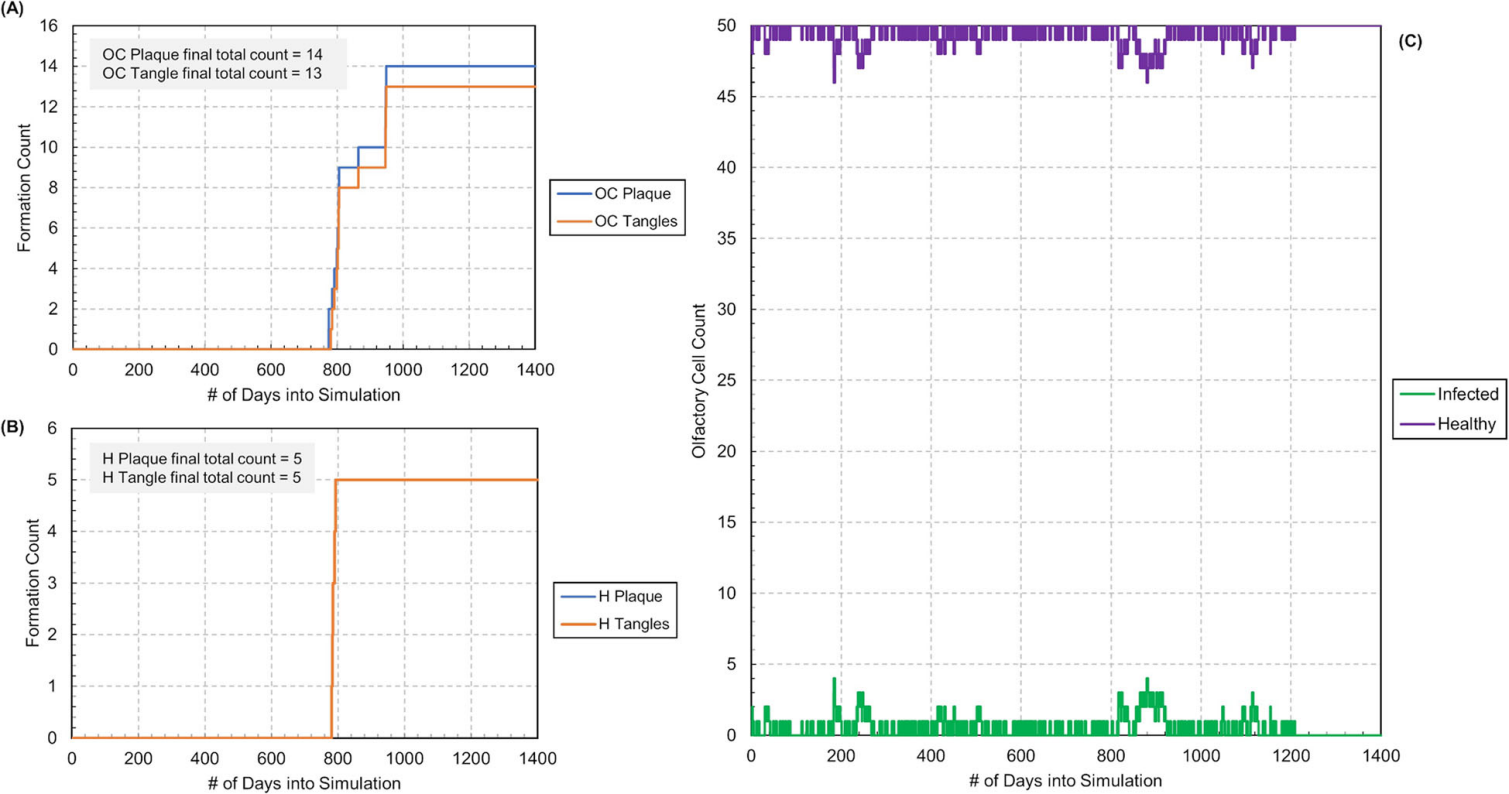
Simulation results for chance of infection of 0.8 (time equivalent ~ 5 years). Infection ends after approximately 1200 days. **a** Plot of Aβ plaque and NF tangle counts in the olfactory cortex (OC) versus simulation run time in days. **b** Plot of Aβ plaque and NF tangle counts in the hippocampus (H) versus simulation run time in days. **c** Plot of infected and healthy olfactory cell counts in the olfactory epithelium versus simulation run time in days

We then tested the effects of changing the probability a patient would get the infection. Using the simulation in Fig. 4 for comparison, the “chance of infection” variable was adjusted to 0.8 as shown in Fig. 6. This may represent an older individual with a healthier immune system. The simulation demonstrated that the overall formation counts were significantly reduced. The Aβ plaque and NF tangle formation counts in the lower infection chance simulation were between one half to one third of those in the higher infection chance simulation. Interestingly, we also observed that that the infection chance played a significant role in determining when the first Aβ plaque appeared. With the higher infection chance, this first Aβ plaque formed around 1 year into the simulation, however with the lower infection chance, the first Aβ plaque formed around 2 years into the simulation. Additionally, although the simulations demonstrated similar olfactory health in the simulation beginning, the body fought off the infection completely in the lower infection chance simulation. This is seen as the olfactory healthy graph hit an absolute steady state and all olfactory epithelium cells remain healthy for the rest of the simulation run. This may be because the body has time to be able to fight off the bacterial infection, as there is a lower chance of infection, before further infection propagation. Nevertheless, the significance of this variable may be useful to test in further model and experiment testing.

Table 5 lists the initial conditions under which the simulation in Fig. 6 was run. All other settings not listed in this table (e.g. olfactory cell tails, bacteria tails, *C. pneumoniae* exposure) were held constant between each trial.

**Table 5:** Settings of user selected parameters for altered infection chance simulation (Fig. 6)

Attribute	Fig 6 Values
EB Concentration	100
Initial Number of Olfactory Cells	50
Initial Number of Sustentacular Cells	50
Initial Number of Mitral Cells	15
Initial Number of Microglia (Olfactory Bulb)	15
Initial Number of Olfactory Cortex Neurons	30
Initial Number of Microglia (Olfactory Cortex)	15
Initial Number of Hippocampus Neurons	30
Initial Number of Microglia (Hippocampus)	15
**Chance of Infection**	**0.8**
Microglia Chance of Healing	0.2
Olfactory Recovery Ability	0.3

## Discussion

### Model implications

Outside of behavior prediction and data fitting, there are several ways our model may be utilized to understand and test the implications of the hypotheses discussed [28]. The model organizes the available information into a visual form, which can help better understand and communicate the mechanics of the considered pathway. It describes a highly dynamic, regulatory and sophisticated system that would be very difficult to understand if taken as a whole. Through simplification, it is much easier to reason and understand the principles behind the system for later experimental design. Since a lot of interactions in the body rely on temporal and spatial variables, the use of NetLogo 3D allows inclusion of time (via its ticks) and spatial interactions (via its visual view), for better description of the system. Additionally, our model clearly shows a time delay between bacterial infection and Aβ plaque production, guiding experimental design to test the proposed mechanism. Finally, our model can play a key role in the testing of other hypotheses which initiate Aβ plaque formation. Through this hypothesis testing, the current set of hypotheses can be refined, discriminated and removed as seen fit to better understand AD etiology.

As with most models, particularly those attempting to represent complex biological systems, limitations may exist to which one can analyze or predict in vivo behavior due to the many simplifying assumptions that were made in modelling the system. In other words, not all published data on the mechanism behind AD and C. pneumoniae exposure was included in the model development as the objective was to create an initial modelling framework to build upon with future available research. For instance, in our model, the movement of EBs within the nasal cavity was adjusted to conform to the software language but might not necessarily meet their true fluid motion when they are breathed in from the environment. In addition, to reduce system complexity, the model does not attempt to include all cell types present, such as astroglia, but rather selectively involves a group of potential main players to represent fundamental actions of the larger community in AD pathogenesis. Instead, for example, neurons served as the main target of infection since they are primary constituents of the hippocampus [40]. Astroglia were not included in the modelling framework on the grounds of system simplification.

In addition, many biological phenomena, such as microbial persistence, were not captured by included model functions. Persistence is a non-genetic, non-inherited ability displayed by bacteria to tolerate certain stresses. This distinct and dormant physiological state often enables long-term survival of a small fraction of the bacterial population leading to chronic and/or recurrent infection [41]. Since the fraction of persisting cells in each population varies greatly by strain and environment, and the molecular mechanisms behind persistence for the pathway considered are not firmly understood yet, no function was created specifically to represent this behavior in the model. In vivo, the existence of persistence can significantly alter the temporal progression of infection, thus confounding predictions of disease development. As more information becomes available, our model can be updated to better represent these true physiological behaviors. This idea of validation by comparing or fitting experimental data is a common step in computational modeling [28]. Our hope is that the continued research of the *C. pneumoniae*-Olfactory mechanism and the system it affects reveals further insight into these values, at which point the model can be easily adapted to fit the findings. Hence, our model remains flexible for the user in case any changes are included. All variables considered are globalized and are provided on the program interface for users to adjust accordingly. For example, in the case a user would like to model exposure by a different single microorganism [9], they can encode the key properties of the organism (e.g. size, environmental concentration, replication rate or incubation time) into the code in a similar manner and adjust or duplicate the given commented user interface variables to create the desired simulation. It may also be necessary to include additional interactions between newly included agents and the environment to explore and compare effects. Our NetLogo model is extremely beneficial to use in this situation as the software is easily accessible and the underlying code has been shared. It is also easy to learn to use as the work has been heavily commented. Additionally, while some researchers have encountered difficulties in isolating *C. pneumoniae* from AD patients, this may to be due to differences in experimental techniques or the likelihood that there is more than one underlying cause of AD [12, 42, 43] Therefore, this model may address only a portion of those who suffer from the disease or a portion of the mechanistic pathways that might occur.

### Recommendations for future work

There are a few different approaches that can be considered to improve the model developed in this paper. First, there are improvements to the NetLogo model that could be made since there were many simplifying assumptions used to develop the model from a programming perspective. Certain areas of code improvement might include adjusting the EB movement to exhibit more realistic behavior, improving axonal depiction, and improving the infection mechanism of *C. pneumoniae*. We also only considered a single dosing scenario for the program, but it may be that multiple inoculations by *C. pneumoniae* – from chronic infection for example – will alter the rates of bacterial propagation and AD symptom development. This is especially so considering, with the current model, a change in the amount of *C. pneumoniae* flux drastically changed the numbers and times of first formation of Aβ plaque and NF tangles. This should be adjustable in our model in the case of pulsing microbial fluxes into the model with the switch for *C. pneumoniae* exposure on the user interface. However, more information may be needed to determine more accurate induction and temporal progression between inoculations. When more research into the effects and values of these related parameters for a multiple dosing scenario are available, it may be an interesting next step to incorporate this information into the model. Once more parameters are identified for *C. pneumoniae* infection in the brain through research, the model can be improved to represent the infection with higher accuracy.

Additionally, the actual mechanism presented by the model may be extended and expanded upon. This model only touches upon a possible precursor to the development of AD, as an initial trigger to Aβ plaque production. Therefore, it may also be beneficial to model the effects of how Aβ plaque deposition may trigger other events in AD pathogenesis. The role of lipopolysaccharides in the inflammatory response can also be explored [11]. Additionally, for the purposes of our model, we looked at *C. pneumoniae* propagation through direct infection of olfactory epithelial cells as a simplifying assumption based on the infection mechanism proposed by the mouse studies of Little et al. An alternative mode of infection, which was considered but not included directly in the model, involves shuttling via host immune cells. Direct shuttling through the blood brain barrier via macrophage recruitment after an inflammatory stimulus was not considered in our model for simplification purposes.. *C. pneumoniae* has been shown to also cause respiratory infection wherein the organism can directly infect lung epithelial cells and alveolar macrophages and then disseminate to nearby leukocytes such as monocytes, lymphocytes and neutrophils [44]. Chronic infection can subsequently lead to systematic infection, through the blood, and the organism can make its way to distal regions of the olfactory epithelium [44]. Research has also shown that leukocytes can bypass the blood brain barrier and move into the central nervous system by passing through the olfactory epithelium and into the olfactory bulbs [45]. In this case, the developed NetLogo model does not comprehensively capture this indirect introduction of the pathogenic microbe to the olfactory system. However, the model may still predict microbial propagation into the brain. As a result, it would be interesting to research and model the combined infection of olfactory epithelial cells and leukocytes, in the future, which may both lead to bacterial propagation into the brain.

Furthermore, studies have drawn a link between genetic risk factors and *C. pneumoniae* load in the central nervous system. The E4 allele of the APOE gene is currently the strongest risk factor for late-onset AD [46]. This gene, expressed extensively by astrocytes in the brain, encodes a protein component of the low-density lipoprotein (LDL) which transports cholesterol to neurons by binding to several cell surface receptors [25]. Studies have shown that AD patients with the E4 allele, which has also been implicated in the development of atherosclerosis and coronary heart disease, display significantly higher numbers of *C. pneumoniae* infected cells in affected brain regions compared to congruent samples from AD patients lacking the allele [47]. Further work by Gerard et al. demonstrates that the APOE E4 can bind to chlamydial EBs while maintaining the ability to bind to LDL receptors on the surface of host sells [48]. The expression of the E4 allele leads to a several fold increase in transport and adherence of EBs of *C. pneumoniae* to astroglia and microglia in vitro [48]. As research progresses and the mechanism is further studied, the NetLogo modelling framework may be updated to explore the difference in infection dynamics between E4 allele carriers and non-carriers.

Additionally, better ways to isolate microbes in brain samples is required to have more data for model validation as current methods may be largely invasive [49]. Most studies performed to allow the model to be developed thus far were performed on deceased patient samples. Nevertheless, it is extremely difficult to isolate *C. pneumoniae* from the brain. Numerous papers have expounded on this difficulty, especially in the reproducibility of studies done by researchers [25]. However, there is a definite need for ethical, longitudinal studies to understand the proposed pathway and its mechanistic parameters better. These parameters include more accurate lysis and *C. pneumoniae* cell cycle lifespans in the brain and a threshold for how long an immune response needs to be sustained to cause Aβ plaque or NF tangle deposition or prolonged inflammation. A verification of the mode of infection would also improve the model. While there are studies which analyze *C. pneumoniae* infections in other parts of the body, such as the lungs, it would be useful to ensure that the infection acts the same in both organs. Strains of *C. pneumoniae* that infect the brain differ from infections in the respiratory tract, thus expounding on the need for experiments to understand infection in the brain [10]. However, our model ultimately lays the foundation for understanding one possible pathway of the *C. pneumoniae*-Olfactory mechanism and provides guidance for these types of experimental tests.

## Conclusion

We have created an initial modeling framework that maps how microbes may propagate through the olfactory system and play a role in the development and progression of Alzheimer’s disease using NetLogo 3D. Our agent-based model simulates the propagation of bacterial infection through the olfactory tract and into the olfactory cortex and hippocampus regions of the brain. By incorporating spatial and temporal behaviors, the model attempts to demonstrate how complex mechanisms may underlie AD pathogenesis. As seen through initial model testing and the simulations described in this paper, given sporadic exposure to *C. pneumoniae*, older individuals or individuals with waning immune systems developed Aβ plaque or NF tangles in the olfactory cortex and hippocampus regions of their brain. These regions have often been cited to possibly be the first areas of deterioration in late-onset AD. Younger individuals with very robust immune systems may not have Aβ plaque or NF tangles at all. Overall, our simulations highlight the two major benefits of modelling this system as an agent-based system with tunable variables: (1) simulations could be adjusted as desired to investigate the effects of different mechanistic parameters and (2) although simulations, with the same initial conditions, had differing specific results, the overall system behavior remained predictable and consistent. While the model may provide a simplified view of the complex system, its results align with developments found in early stages of late-onset Alzheimer’s disease. As a result, we hope the framework can be used and refined for a variety of different purposes such as hypothesis testing and experimental guidance.

## Data Availability

The NetLogo modelling environment software is available for downloading at https://ccl.northwestern.edu/netlogo/ [50]. The datasets generated and/or analyzed during the current study are available in the GitHub repository, https://github.com/shalini-sundar/ADAgentBasedModel. This includes the Alzheimer’s Disease NetLogo model developed in this paper, its documentation and raw data of simulation outputs (*S1 ConstantFlux_Simulation_RawData.zip*, *S2 LowerImmunity_RawData.zip*, *S3 HigherImmunity_Simulation_RawData.zip* and *S4 LowerInfectionChance_Simulation_RawData.zip*).

## References

[1] Alzheimer’s Association (2019). 2019 Alzheimer’s Disease Facts and Figs. Alzheimer’s Association.

[2] DeKosky ST (2001). Epidemiology and pathophysiology of Alzheimer's disease. Clin Cornerstone.

[3] NIH Fact Sheets. Alzheimer’s Disease: NIH; 2018. Available from: https://report.nih.gov/NIHfactsheets/ViewFactSheet.aspx?csid=107. [cited 12 Aug 2019].

[4] HHS Press Office. Obama administration presents national plan to fight Alzheimer’s disease: NIH National Institute in Aging; 2012. Available from: https://www.nia.nih.gov/news/obama-administration-presents-national-plan-fight-alzheimers-disease. [cited 12 Aug 2019].

[5] Pimplikar SW (2009). Reassessing the amyloid cascade hypothesis of Alzheimer’s disease. Int J Biochem Cell Biol.

[6] Behl C (2012). Brain aging and late-onset Alzheimer's disease: many open questions. Int Psychogeriatr.

[7] Lannfelt L (1997). The genetics and pathophysiology of Alzheimer’s disease. J Intern Med.

[8] Tanzi RE, Bertram L (2005). Twenty years of the Alzheimer’s disease amyloid hypothesis: a genetic perspective. Cell..

[9] Itzhaki RF, Lathe R, Balin BJ, Ball MJ, Bearere EL, Braak H (2016). Microbes and Alzheimer’s disease. J Alzheimers Dis.

[10] Dreses-Werringloer U, Bhuiyan M, Zhao Y, Gérard HC, Whittum-Hudson JA, Hudson AP (2009). Initial characterization of Chlamydophila (chlamydia) pneumoniae cultured from the late-onset Alzheimer brain. Int J Med Microbiol.

[11] Little CS, Hammond CJ, MacIntyre A, Balin BJ, Appelt DM (2004). Chlamydia pneumoniae induces Alzheimer-like amyloid plaques in brains of BALB/c mice. Neurobiol Aging.

[12] Contini C, Seraceni S, Cultrera R, Castellazzi M, Granieri E, Fainardi E. Chlamydophila pneumoniae infection and its role in neurological disorders. Interdiscip Perspect Infect Dis. 2010. 10.1155/2010/273573. 10.1155/2010/273573 PMC2825657 20182626

[13] Appelt D, Cappellini C, Cader A (2013). Changes in calcium-related gene expression consistent with Alzheimer’s disease are initiated by infection of neuronal cells with chlamydia pneumoniae. Alzheimers Dement.

[14] Sundar S, McNulty R, Battistoni C, Morales F. Agent Based Model for Late-Onset Alzheimer’s Disease: GitHub. GitHub Inc 2019; 2019. Available from: https://github.com/shalini-sundar/ADAgentBasedModel. [cited 2 September 2019].

[15] Tang J, Alsop RJ, Backholm M, Dies H, Shi AC, Rheinstädter MC (2016). Amyloid-β 25–35 peptides aggregate into cross-β sheets in unsaturated anionic lipid membranes at high peptide concentrations. Soft Matter.

[16] Raj A, Kuceyeski A, Weiner M (2012). A network diffusion model of disease progression in dementia. Neuron..

[17] Puri IK, Li L. Mathematical Modeling for the Pathogenesis of Alzheimer's Disease. PloS One. 2010;5(12). 10.1371/journal.pone.0015176. 10.1371/journal.pone.0015176 PMC3001872 21179474

[18] CDC. Chlamydia pneumoniae Infection Signs and Symptoms: CDC; 2019. Available from: https://www.cdc.gov/pneumonia/atypical/cpneumoniae/about/symptoms.html. [cited 12 Aug 2019].

[19] Rupp J, Pfleiderer L, Jugert C, Moeller S, Klinger M, Dalhoff K, et al. Chlamydia pneumoniae Hides inside Apoptotic Neutrophils to Silently Infect and Propagate in Macrophages. PLoS One. 2009;4(6). 10.1371/journal.pone.0006020. 10.1371/journal.pone.0006020 PMC2695784 19547701

[20] Byrne GI, Ouellette SP, Wang Z, Rao JP, Lu L, Beatty WL (2001). Chlamydia pneumoniae expresses genes required for DNA replication but not cytokinesis during persistent infection of HEp-2 cells. Infect Immun.

[21] Balin BJ, Gérard HC, Arking EJ, Appelt DM, Branigan PJ, Abrams JT (1998). Identification and localization of chlamydia pneumoniae in the Alzheimer’s brain. Med Microbiol Immunol.

[22] Gérard HC, Dreses-Werringloer U, Wildt KS, Deka S, Oszust C, Balin BJ (2006). Chlamydophila (chlamydia) pneumoniae in the Alzheimer’s brain. FEMS Immunol Med Microbiol.

[23] Hammond CJ, Hallock LR, Howanski RJ, Appelt DM, Little CS, Balin BJ (2010). Immunohistological detection of chlamydia pneumoniae in the alzheimer's disease brain. BMC Neurosci.

[24] Kumar DK, Choi SH, Washicosky KJ (2016). Amyloid-β peptide protects against microbial infection in mouse and worm models of alzheimer's disease. Sci Transl Med.

[25] Balin BJ, Hammond CJ, Little CS, Hingley ST, Al-Atrache Z, Appelt DM, et al. Chlamydia pneumoniae: an etiologic agent for late-onset dementia. Front Aging Neurosci. 2018;10. 10.3389/fnagi.2018.00302. 10.3389/fnagi.2018.00302 PMC6189393 30356749

[26] Mann DM, Tucker CM, Yates PO (1988). Alzheimer’s disease: an olfactory connection?. Mech Ageing Dev.

[27] Christen-Zaech S, Kraftsik R, Pillevuit O, Kiraly M, Martins R, Khalili K (2003). Early olfactory involvement in Alzheimer’s disease. Can J Neurol Sci.

[28] Dhurjati P, Mahadevan R (2008). Systems biology: the synergistic interplay between biology and mathematics. Can J Chem Eng.

[29] Jafek BW, Murrow B, Michaels R, Restrepo D, Linschoten M, Taste RM (2002). Biopsies of human olfactory epithelium. Chemical Sense.

[30] Kavoi BM, Jameela H (2011). Comparative morphometry of the olfactory bulb, tract and stria in the human, dog and goat. Int J Morphol.

[31] Panhuber H, Laing DG (1987). The size of mitral cells is altered when rats are exposed to an odor from their day of birth. Brain Res Dev Brain Res.

[32] Nimmerjahn A, Kirchhoff F, Helmchen F (2005). Resting microglial cells are highly dynamic Surveillants of brain parenchyma in vivo. Science..

[33] Little CS, Joyce TA, Hammond CJ (2014). Detection of bacterial antigens and alzheimer's disease-like pathology in the central nervous system of balb/c mice following intranasal infection with a laboratory isolate of chlamydia pneumoniae. Front Aging Neurosci.

[34] Morrison J (2014). The sense of SMELL; 2014 [cited 12 Aug 2019]. In: HumanPhysiology. Academy [internet].

[35] Sultan-Styne K, Toledo R, Walker C, Kallkopf A, Ribak CE, Guthrie KM (2009). Long-term survival of olfactory sensory neurons after target depletion. J Comp Neurol.

[36] Price JL, Squire LR (2009). Olfactory higher centers anatomy. Encyclopedia of neuroscience.

[37] Simpson KL, Haines DE, Gregory A (2018). Olfaction and Taste. Mihailoff. Fundamental Neuroscience for Basic and Clinical Applications.

[38] Frost GR, Li YM. The role of astrocytes in amyloid production and Alzheimer’s disease. Open Biol. 2017;7(12). 10.1098/rsob.170228. 10.1098/rsob.170228 PMC5746550 29237809

[39] Oddo S, Caccamo A, Kitazawa M, Tseng BP, LaFerla FM (2003). Amyloid deposition precedes tangle formation in a triple transgenic model of Alzheimer’s disease. Neurobiol Aging.

[40] von Bartheld CS, Bahney J, Herculano-Houzel S (2016). The search for true numbers of neurons and glial cells in teh human brain: a review of 150 years of cell counting. J Comp Neurol.

[41] Jung S, Ryu C, Kim J (2019). Bacterial persistence: fundamentals and clinical importance. J Microbiol.

[42] Taylor GS, Vipond IB, Paul ID, Matthews S, Wilcock GK, Caul EO (2002). Failure to correlate C. pneumoniae with late onset Alzheimer’s disease. Neurology..

[43] Balin BJ, Hudson AP. Etiology and pathogenesis of late-onset Alzheimer’s disease. Curr Allergy Asthma Rep. 2014. 10.1007/s11882-013-0417-1. 10.1007/s11882-013-0417-1 24429902

[44] Porritt RA, Crother TR (2016). Chlamydia pneumonaie infection and inflammatory diseases. Innunopathol Dis Therap.

[45] Getchell TV, Subhedar NK, Shah DS (2002). Chemokine regulation of macrophage recruitment into the olfactory epithelium following target ablation: involvement of macrophage inflammatory protein-1α and monocyte chemoattractant protein-1. J Neurosci Res.

[46] Liu CC, Liu CC, Kanekiyo T, Xu H, Bu G (2013). Apolipoprotein E and Alzheimer disease: risk, mechanisms and therapy. Nat Rev Neurol.

[47] Gérard HC, Wildt KL, Whittum-Hudson J, Lai Z, Ager J, Hudson AP (2005). The load of chlamydia pneumoniae in the Alzheimer’s brain varies with APOE genotype. Microb Pathog.

[48] Gérard HC, Fomicheva E, Whittum-Hudson J, Hudson AP (2008). Apolipoprotein E4 enhances attachment of Chlamydophila (chlamydia) pneumoniae elementary bodies to host cells. Microb Pathog.

[49] Dominy SS, Lynch C, Ermini F, Benedyk M, Marczyk A, Konradi A, et al. Porphyromonas gingivalis in Alzheimer’s disease brains: Evidence for disease causation and treatment with small-molecule inhibitors. Sci Adv. 2019;5(1). 10.1126/sciadv.aau3333.. 10.1126/sciadv.aau3333 PMC6357742 30746447

[50] Wilensky U (1999). NetLogo: Agent-Based Modelling Software. Version 6.1.0 [software]. Northwestern University.

